# *In vivo*, genome-wide profiling of endogenously tagged chromatin-binding proteins with spatial and temporal resolution using NanoDam in *Drosophila*

**DOI:** 10.1016/j.xpro.2022.101788

**Published:** 2022-11-01

**Authors:** Jocelyn L.Y. Tang, Robert Krautz, Oriol Llorà-Batlle, Anna E. Hakes, Paul M. Fox, Andrea H. Brand

**Affiliations:** 1 Gurdon Institute and Department of Physiology, Development and Neuroscience, University of Cambridge, Tennis Court Road, Cambridge CB2 1QN, UK

**Keywords:** Cell biology, Developmental biology, Genomics, Sequencing, Model organisms

## Abstract

NanoDam is a technique for genome-wide profiling of the binding targets of any endogenously tagged chromatin-binding protein *in vivo*, without the need for antibodies, crosslinking, or immunoprecipitation. Here, we explain the procedure for NanoDam experiments in *Drosophila*, starting from a genetic cross, to the generation of sequencing libraries and, finally, bioinformatic analysis. This protocol can be readily adapted for use in other model systems after simple modifications.

For complete details on the use and execution of this protocol, please refer to Tang et al. (2022).

## Highlights

• NanoDam identifies genome-wide binding targets of endogenously-tagged proteins
• NanoDam profiling in *Drosophila* can be achieved in a single genetic cross
• Protocol for performing a NanoDam experiment from sample collection to data analysis
• Protocol can be adapted for a variety of model systems

## Abstract (editor highlights)

Publisher’s note: Undertaking any experimental protocol requires adherence to local institutional guidelines for laboratory safety and ethics.

## Graphical abstract

**Figure:**
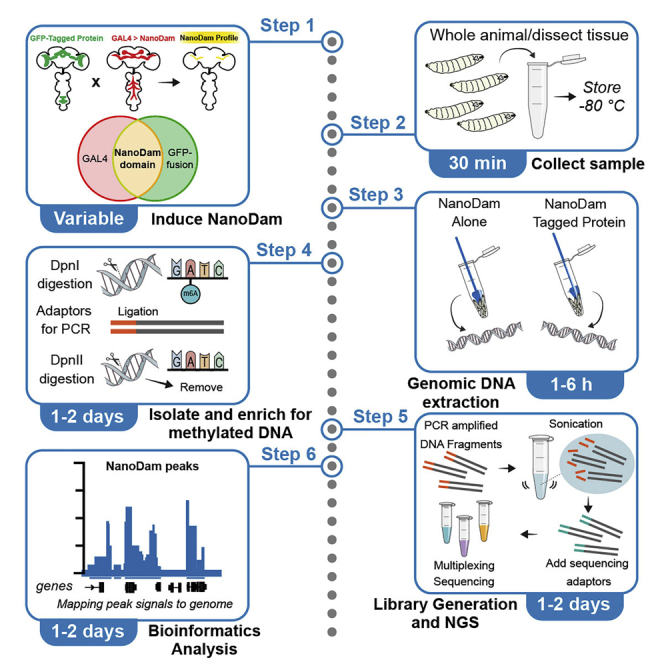

## Before you begin

Profiling the interaction of proteins with chromatin *in vivo* is an essential step in understanding how gene regulation affects biological functions in different cell types. Although chromatin immunoprecipitation (ChIP) is commonly used, it depends upon the availability of specific antibodies. Furthermore, cell-type specificity has to be achieved through careful cell isolation (via fluorescent-activated cell sorting (FACS). As an alternative approach, van Steensel and Henikoff (2000) developed DNA adenine methyltransferase identification (DamID). DamID uses a Dam methylase (from *E. coli*) fused to a protein of interest to methylate GATC sites neighboring the protein of interest’s binding sites. Building upon this, targeted DamID (TaDa) (Southall et al., 2013) was developed to place DamID under the control of the GAL4 system (Brand and Perrimon, 1993), which enables *in vivo* profiling and cell-type specificity without cell isolation. With TaDa, transgenes are first generated (i.e., the protein of interest fused to Dam methylase under UAS control) and the Dam fusion protein is expressed from a bicistronic mRNA, enabling low level expression and circumventing Dam-associated toxicity. To simplify TaDa and avoid the need for transgenic constructs, we developed NanoDam (Tang et al., 2022). NanoDam profiles the binding targets of endogenous proteins *in vivo*, with both spatial and temporal specificity. NanoDam makes use of a nanobody, a recombinant single-domain antibody originally found in camelid species, distinguished from conventional antibodies by their small size, higher stability and solubility (Muyldermans, 2001). The nanobody is used to target Dam methylase to a chromatin binding protein tagged with GFP, or any other tag recognized by a specific nanobody. Restricting the expression of NanoDam with the GAL4 system enables the genome-wide binding profile of any tagged factor to be investigated in a defined subset of its endogenous expression pattern after a single genetic cross.

The steps below describe how to perform a NanoDam experiment in *Drosophila*, with the use of the GAL4 system for cell-type-specific expression and a temperature-sensitive GAL80 (tubGAL80^ts^) to control the timing of GAL4 expression. However, steps from genomic DNA extraction onwards can be applied to other organisms, allowing NanoDam experiments to be applied to other model systems (e.g., organoids, cell cultures). The transgenic tools for spatial and temporal specific induction of NanoDam will vary depending on the organism and technology available. Endogenously-tagged proteins can be sourced either from stock centers (in the case of *Drosophil*a for example) or generated via CRISPR/Cas9 genome editing.

### Institutional permissions

Ensure that all experiments performed adhere to the relevant regulatory standards or national guidelines and permission has been acquired from the relevant institutions.

### NanoDam induction *in vivo* and tissue isolation

**Timing: variable; dependent on tissue of interest and developmental stage**

This is the profiling step of the experiment. NanoDam is expressed in the cell types of interest by GAL4 driver. If the endogenously tagged protein of interest is also present in these cell types, NanoDam will methylate the neighboring GATC sequences depending on where the protein binds in the genome.

**Figure 1:**
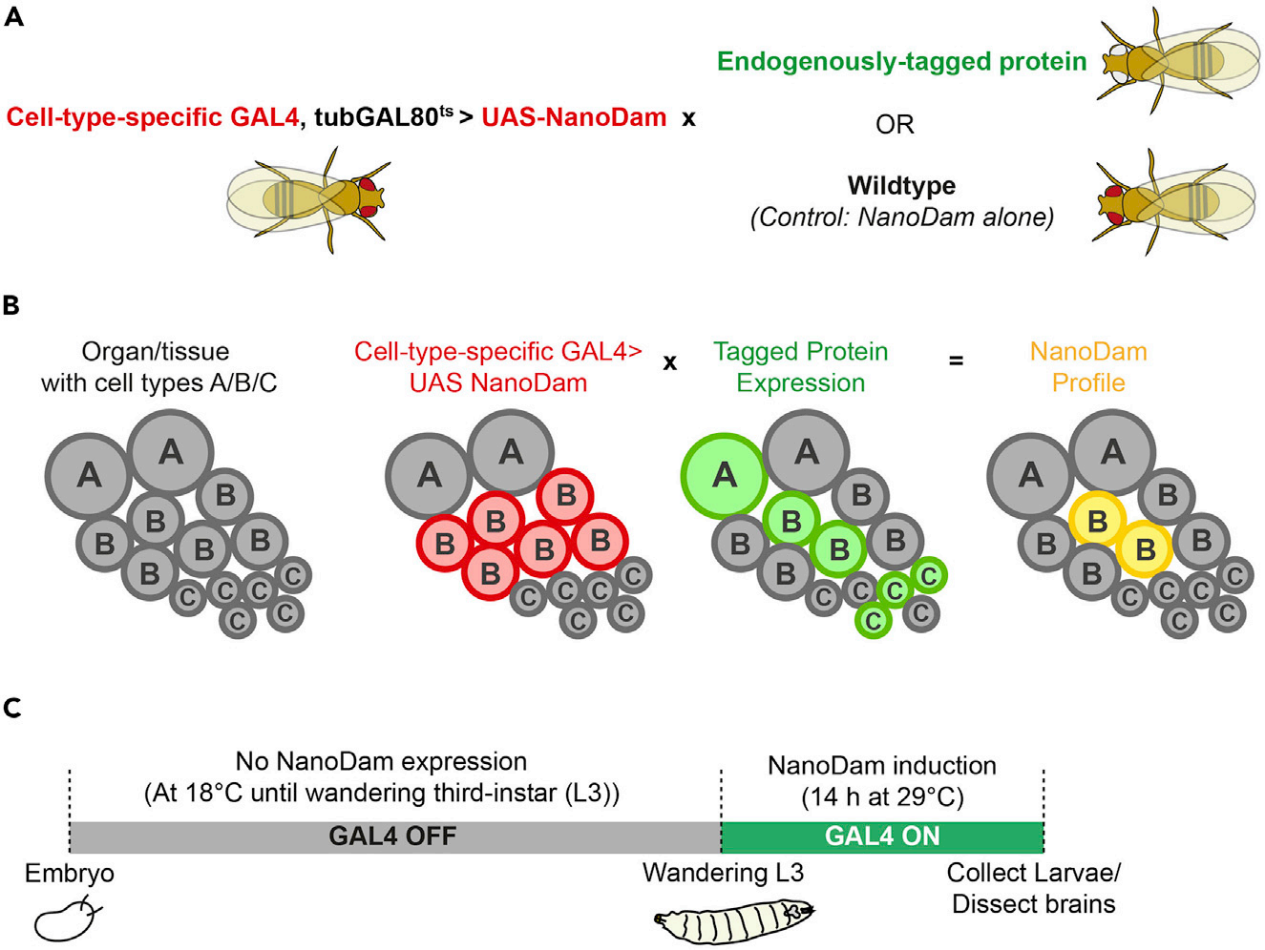
Example of a NanoDam experiment in *Drosophila* (A) Genetic cross set up. (B) Diagram of how high spatial and temporal resolution can be achieved with NanoDam. (C) Example of the timeline of NanoDam induction, if cells of interest were to be profiled at wandering third-instar (L3).
2. Induce NanoDam for a minimum of 10 h prior to the timepoint of interest, by shifting from 18°C (GAL4 expression inhibited) to 29°C.

***Note:*** Typical induction times for NanoDam range from 10 h to 14 h. These times depend on the expression of the protein and cell types of interest.

1. Set up a genetic cross with GAL4>UAS-NanoDam with the endogenously-tagged protein of interest. (See Figure 1 for example).
  a. For the control, set up a cross with GAL4>UAS-NanoDam in the absence of the tagged protein (ideally using a fly line with the same genetic background as the experimental condition).
  b. Use the temperature-sensitive tubGAL80^ts^ to restrict the expression of the GAL4 to the time frame needed.

3. Isolate the tissue of interest by dissection or other appropriate methods. If not required, collect whole animals (e.g., larvae) and place them into a 1.5 mL Eppendorf tube.
  a. Dissect tissues in PBS.
  b. Remove excess PBS with a pipette and store tissue until required.

**Pause point:** Tissue can be stored at −20°C or at −80°C.

***Note:*** First-instar and third-instar whole larvae have also been tried with successful results (in the context of profiling binding in neural stem cells and avoiding dissection). However, this depends on specific experimental setup, such as the specificity of the GAL4 driver (for the cell types of interest), the protein of interest being profiled and the numbers of cells per organism. If cells of interest are rare, tissue can be stored long-term and processed once sufficient amounts have been collected. Replicates can also be stored and processed simultaneously. Aim for 3 biological replicates per condition (including NanoDam alone).

### Sera-mag bead preparation

**Timing: 30 min**

This preparation step for Sera-mag beads was modified from (Rohland and Reich, 2012). These beads will later be used for multiple rounds of DNA purification.

***Optional:*** Using homebrew Sera-mag beads is a cost-effective way to perform this protocol. However, this step can be skipped if commercial alternatives are used (see materials and equipment below).

4. In a 15 mL Falcon tube add:

**Table:** 

Reagent	Final concentration	Amount
PEG-8000	20% w/v	3 g
NaCl (5 M)	1 M	3 mL
Tris-HCl pH8.0	10 mM	150 μL
EDTA (0.5 M)	1 mM	30 μL
dH_2_O	N/A	Up to 14 mL
**Total**	**N/A**	**14 mL**

**CRITICAL:** Ensure the 3 g of PEG-8000 is weighed accurately as this will impact the size of DNA fragments that will be Isolated.

5. Mix by inverting the tube up and down until the PEG is dissolved (∼5 min).
6. Mix Sera-mag Speedbeads stock well to ensure beads are in suspension.
7. Add 300 μL Sera-mag Speedbeads and mix by inverting the tube.
8. Add dH_2_O up to a 15 mL total volume and store 4°C.
9. Test beads by purifying the Bioline Hyperladder (25 bp ladder).
  a. Make master mix of diluted ladder by mixing 10 μL ladder and 20 μL dH_2_O per sample.
  b. Purify ladder with 1×, 1.25× and 1.5× amount of beads.
    i. Add required volume of beads to 30 μL of sample and mix well. Vortex and pulse down for 5 s. Rapid mixing of beads and sample is crucial.
    ii. Incubate at 21°C–24°C for 10 min,
    iii. Place on magnetic stand for 2 min or until the solution is clear.
    iv. Discard the supernatant.
    v. Wash for 30 s with 190 μL 80% ethanol/dH_2_O. Perform 2 washes.
    vi. Let samples stand for 1 min to air dry (avoid over-drying). A P10 pipette can be used to remove the last drops of 80% ethanol.
    vii. Resuspend in 20 μL dH_2_O and remove from the magnetic stand.
    viii. Mix well by vortexing and incubate for 2 min at 21°C–24°C.
    ix. Place on the magnetic stand for 2 min or until the solution is clear.
    x. Put 20 μL of the supernatant into a new, clean PCR tube.
  c. Run on the Tapestation using D1K tape.

***Note:***Rohland and Reich (2012) diluted Sera-mag beads in 18% PEG-8000 but for the purposes of NanoDam, dilution in 20% PEG-8000 is better and gives comparable results to Agencourt Ampure XP beads (commercial alternative).

### dsAdR stock for adaptation ligation buffer

**Timing: 1–2 h (For step 16)**

**Figure 2:**
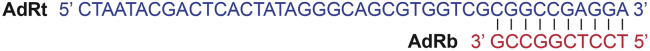
Diagram of the annealed structure of dsAdR

Make a 50 μM AdR stock by annealing DamID adaptors (Figure 2).

10. Take 50 μL AdRt (100 μM in dH_2_O) and 50 μL AdRb (100 μM in dH_2_O).
11. Incubate in removable metal heating block at 95°C for 2 min.
12. Remove heating bock and allow to cool to 21°C–24°C.
13. Store the dsAdR stock at −20°C for up to 6 months.

### Buffer and reagent preparation

There are several homebrew buffers in this protocol that can be made in advance and stored at −20°C before use. Making the buffers before beginning greatly increases the efficiency and ease of following this protocol. (See the materials and equipment section below for details on the reagents needed for each buffer.).

14. Make adaptor ligation buffer (for step 16) using the dsAdR stock generated previously.
15. Make DpnII digestion buffer (for step 18).
16. Make MyTaq Master mix (for step 31).
17. Make resuspension buffer (for steps 41, 43, 47, 49).
18. Make 10 mM dNTP mix and end repair buffer (for step 44).
19. Make End repair enzyme mix (for step 44).
20. Make NGS PCR primer mix (for step 48).
21. Store all mixes at −20°C for 6 months–1 year.

### Annealing of NGS adaptors

**Timing: 1–2 h (For step 46)**

22. Resuspend adaptor oligos at 100 μM in TE.
23. Mix 25 μL of relevant index adaptor (1–19) and 25 μL of Universal Primer. (See Table 1).

**Table 1:** List of sequencing adaptors

Adaptor	Sequence
Universal	AATGATACGGCGACCACCGAGATCTA CACTCTTTCCCTACACGACGCTCTTCCGATC∗T
Index 1	[Phos]GATCGGAAGAGCACACGTCTGAACTCCAGTCAC **ATCACG**ATCTCGTATGCCGTCTTCTGCTT∗G
Index 3	[Phos]GATCGGAAGAGCACACGTCTGAACTCCAGTCAC **TTAGGC**ATCTCGTATGCCGTCTTCTGCTT∗G
Index 8	[Phos]GATCGGAAGAGCACACGTCTGAACTCCAGTCAC **ACTTGA**ATCTCGTATGCCGTCTTCTGCTT∗G
Index 9	[Phos]GATCGGAAGAGCACACGTCTGAACTCCAGTCAC **GATCAG**ATCTCGTATGCCGTCTTCTGCTT∗G
Index 10	[Phos] GATCGGAAGAGCACACGTCTGAACTCCAGTCAC **TAGCTT**ATCTCGTATGCCGTCTTCTGCTT∗G
Index 11	[Phos]GATCGGAAGAGCACACGTCTGAACTCCAGTCAC **GGCTAC**ATCTCGTATGCCGTCTTCTGCTT∗G
Index 13	[Phos]GATCGGAAGAGCACACGTCTGAACTCCAGTCAC **AGTCAA**ATCTCGTATGCCGTCTTCTGCTT∗G
Index 14	[Phos]GATCGGAAGAGCACACGTCTGAACTCCAGTCAC **AGTTCC**ATCTCGTATGCCGTCTTCTGCTT∗G
Index 15	[Phos]GATCGGAAGAGCACACGTCTGAACTCCAGTCAC **ATGTCA**ATCTCGTATGCCGTCTTCTGCTT∗G
Index 18	[Phos]GATCGGAAGAGCACACGTCTGAACTCCAGTCAC **GTCCGC**ATCTCGTATGCCGTCTTCTGCTT∗G
Index 2	[Phos]GATCGGAAGAGCACACGTCTGAACTCCAGTCAC **CGATGT**ATCTCGTATGCCGTCTTCTGCTT∗G
Index 4	[Phos]GATCGGAAGAGCACACGTCTGAACTCCAGTCAC **TGACCA**ATCTCGTATGCCGTCTTCTGCTT∗G
Index 5	[Phos]GATCGGAAGAGCACACGTCTGAACTCCAGTCAC **ACAGTG**ATCTCGTATGCCGTCTTCTGCTT∗G
Index 6	[Phos]GATCGGAAGAGCACACGTCTGAACTCCAGTCAC **GCCAAT**ATCTCGTATGCCGTCTTCTGCTT∗G
Index 7	[Phos]GATCGGAAGAGCACACGTCTGAACTCCAGTCAC **CAGATC**ATCTCGTATGCCGTCTTCTGCTT∗G
Index 12	[Phos] GATCGGAAGAGCACACGTCTGAACTCCAGTCAC **CTTGTA**ATCTCGTATGCCGTCTTCTGCTT∗G
Index 16	[Phos] GATCGGAAGAGCACACGTCTGAACTCCAGTCAC **CCGTCC**ATCTCGTATGCCGTCTTCTGCTT∗G
Index 19	[Phos]GATCGGAAGAGCACACGTCTGAACTCCAGTCAC **GTGAAA**ATCTCGTATGCCGTCTTCTGCTT∗G

[Phos] = 5′ Phosphorylation, ∗ = Phosphorothioate linkages; adaptor barcodes in bold.
24. Add 0.5 μL of 5 M NaCl.
25. Incubate in a removable metal heating block at 95°C for 2 min.
26. Remove heating block and allow to cool to 21°C–24°C.
27. Annealed oligos can be stored at −20°C for up to 6 months.

## Key resources table

**Table:** 

REAGENT or RESOURCE	SOURCE	IDENTIFIER
**Chemicals, peptides, and recombinant proteins**		

10 mM Tris-HCl (pH 8.0)	N/A	N/A
100% ethanol	VWR	Cat#20821.330
AlwI	NEB	Cat#R0513L
Autoclaved dH_2_O	N/A	N/A
Cytiva Sera-mag Speedbeads (Carboxyl magnetic beads, 1 μM, 15 mL)	Fisher Scientific	Cat#09-981-123
Deoxynucleotide (dNTP) Solution Set	NEB	Cat#N0446S
DpnI (+ CutSmart Buffer)	NEB	Cat#R0176
DpnII (+DpnII Buffer)	NEB	Cat#R0543S
EDTA	Sigma-Aldrich	Cat#E6758
Klenow 3′-5′ exo-enzyme	NEB	Cat#M9212L
Klenow Fragment (5 U/μL)	NEB	Cat#M0210S
MyTaq HS DNA Polymerase	Bioline	Cat#BIO-21113
NEBNext High Fidelity 2× PCR Master Mix (6.25 mL)	NEB	Cat#M0541L
PEG-8000	Sigma-Aldrich	Cat#P5413
Phosphate Buffer Saline (1× PBS)	N/A	N/A
RNAseA (DNAse free)	Roche	Cat#11119915001
T4 DNA Ligase (+ 10× Buffer)	NEB	Cat#M0202S
T4 DNA polymerase (3 U/μL)	NEB	Cat#M0203S
T4 polynucleotide kinase	NEB	Cat#M0201S
25 bp Hyperladder	Bioline	Cat#BIO-33057

**Critical commercial assays**		

Genomic DNA ScreenTape Analysis Reagents	Agilent	Cat#5067-5366
QiaAmp DNA Micro Kit	QIAGEN	Cat#56304
Qiagen PCR Purification Kit	QIAGEN	Cat#28106
Qubit dsDNA HS Assay Kit (500 assays, 0.2–100 ng)	Thermo Fisher Scientific	REFQ32854
Quick Ligation Kit	NEB	Cat#M2200S
Reagents for Bioanalyzer: DNA 1000 Kit	Agilent	Cat#5067-1504

**Deposited data**		

NanoDam data (raw and processed files)	Tang et al. (2022)	GEO: GSE190210

**Oligonucleotides**		

**AdRt Primer (unmodified – 0.05 μmol, desalted)** CTAATACGACTCACTATAGGGCAGCGTGG TCGCGGCCGAGGA	Custom	N/A
**AdRb Primer (unmodified – 0.05 μmol, desalted)** TCCTCGGCCG	Custom	N/A
**DamID PCR Primer** GGTCGCGGCCGAGGATC	Custom	N/A
**NGS_PCR1 (∗ = phosphorothioate linkage)** AATGATACGGCGACCACCGA∗G	Custom	N/A
**NGS_PCR2 (∗ = phosphorothioate linkage)** CAAGCAGAAGACGGCATACGA∗G	Custom	N/A

**Experimental Models: Organisms/strains**		

UAS-*NanoDam*	Tang et al. (2022)	N/A

**Recombinant DNA**		

pUAST-mCherry-Dam-vhhGFP4 (pUAST-NanoDam)	Tang et al. (2022)	N/A

**Software and algorithms**		

FastQC (v0.11.5)		https://www.bioinformatics.babraham.ac.uk/projects/fastqc/
Multiqc (v.1.12)	Ewels et al. (2016)	https://multiqc.info/
Cutadapt	Martin (2011)	https://cutadapt.readthedocs.io/en/stable/
Preseq (v.3.1.2)	Daley and Smith (2013)	http://smithlabresearch.org/software/preseq/
Deeptools (v3.5.1)	Ramírez et al. (2016)	https://pypi.org/project/deepTools/
damidseq_pipeline	Marshall and Brand (2015)	https://github.com/AHBrand-Lab/DamID_scripts
slurm workload manager (v15.08.13)	SchedMD	https://slurm.schedmd.com/download.html
bowtie2 (v2.3.4.1)	Langmead and Salzberg (2012)	http://bowtie-bio.sourceforge.net/bowtie2/index.shtml
bedGraphToBigWig (v4)	UCSC	https://www.encodeproject.org/software/bedgraphtobigwig/
MACS2 (v2.1.2)	Zhang et al. (2008)	https://pypi.org/project/MACS2/
Samtools	Li et al. (2009)	http://www.htslib.org
bedtools (v2.26.0)	Quinlan and Hall (2010)	https://github.com/arq5x/bedtools2
Integrative Genomic Viewer (IGV)	Robinson et al. (2011)	https://software.broadinstitute.org/software/igv/download
R	R-project	https://cran.ma.imperial.ac.uk/
RStudio	RStudio	https://www.rstudio.com/products/rstudio/download/
damMer.py	Tang et al. (2022)	https://github.com/AHBrand-Lab/NanoDam_analysis
damMer_tracks.py	Tang et al. (2022)	https://github.com/AHBrand-Lab/NanoDam_analysis
damMer_peaks.py	Tang et al. (2022)	https://github.com/AHBrand-Lab/NanoDam_analysis
genomewide_correlation.Rmd	Tang et al. (2022)	https://github.com/AHBrand-Lab/NanoDam_analysis
signal_enrichment.Rmd	Tang et al. (2022)	https://github.com/AHBrand-Lab/NanoDam_analysis
gatc.track.maker.pl	Marshall and Brand (2015)	https://github.com/AHBrand-Lab/DamID_scripts
quantile_norm_bedgraph.pl		https://github.com/AHBrand-Lab/DamID_scripts
average_tracks.pl		https://github.com/AHBrand-Lab/DamID_scripts

**Other**		

Agilent 2200 Tapestation	Agilent	Cat#G2965AA
Benchtop Centrifuge	Eppendorf	Cat#5424 R
Diagenode Bioruptor Pico (Sonicator)	Diagenode	Cat#B01080010
DynaMag-96side magnet	Invitrogen	Cat#12331D
Genomic DNA ScreenTape	Agilent	Cat#5067-5365
Proflex PCR System	Applied Biosystems	N/A
Qiagen Qubit 3.0	QIAGEN	Cat#Q33216
Quantus Fluorometer	Promega	Cat#E6150
Qubit Assay tubes (500 tubes)	Thermo Fisher Scientific	Cat#Q32856
0.2 mL microtubes for Bioruptor® Pico	Diagenode	Cat#C30010020

## Materials and equipment

**Table:** Adaptor Ligation Buffer (For step 16)

Reagent	Final concentration	Amount
10× T4 DNA Ligase Buffer	5×	200 μL
dsAdR (50 μM)	10 μM	80 μL
dH_2_O	N/A	120 μL
**Total**	**N/A**	**400 μL**

Buffer can be stored as 40 μL aliquots at −20°C for up to 1 year. Avoid freeze/thaw cycles if possible.

**Table:** DpnII digestion buffer (For step 18)

Reagent	Final concentration	Amount
10× DpnII Buffer	2.1×	400 μL
dH_2_O	N/A	1,500 μL
**Total**	**N/A**	**1,900 μL**

Buffer can be stored as 190 μL aliquots at −20°C for up to 1 year. Avoid freeze/thaw cycles if possible.

**Table:** MyTaq Master Mix (For step 31)

Reagent	Final concentration	Amount
5× MyTaq Reaction Buffer	2.6×	1,000 μL
DamID-PCR primer (50 μM)	6.5 μM	250 μL
dH_2_O	N/A	650 μL
**Total**	**N/A**	**1,900 μL**

Buffer can be stored as 190 μL aliquots at −20°C for up to 1 year. Avoid freeze/thaw cycles if possible.

**Table:** Resuspension Buffer (For steps 41, 43, 47, 49)

Reagent	Final concentration	Amount
Tris-HCl pH8.0 (1 M)	10 mM	500 μL
EDTA (0.5 M)	0.1 mM	10 μL
dH_2_O	N/A	49.49 mL
**Total**	**N/A**	**50 mL**

Buffer can be stored at 21°C–24°C.

**Table:** dNTP mix (For End Repair Buffer)

Reagent	Final concentration	Amount
dGTP (100 mM)	10 mM	100 μL
dCTP (100 mM)	10 mM	100 μL
dATP (100 mM)	10 mM	100 μL
dTTP (100 mM)	10 mM	100 μL
dH_2_O	N/A	600 μL
**Total**	**N/A**	**1,000 μL**

Mix can be stored as aliquots at −20°C for up to 1 year. Avoid freeze/thaw cycles if possible.

**Table:** End-Repair Buffer (For step 44)

Reagent	Final concentration	Amount
10× T4 DNA Ligase Buffer	4×	150 μL
dNTPs (10 mM)	1.6 mM	60 μL
dH_2_O	N/A	165 μL
**Total**	**N/A**	**375 μL**

Buffer can be stored as aliquots at −20°C more than 6 months.

**Table:** End-Repair Enzyme Mix (For step 44)

Reagent	Final concentration	Amount
T4 DNA Polymerase (3 U/ μL)	∼1 U/ μL	56.82 μL
Klenow Fragment (5 U/ μL)	∼1 U/ μL	11.36 μL
T4 polynucleotide kinase (10 U/ μL)	∼5 U/μL	56.82 μL
**Total**	**N/A**	**125 μL**

Enzyme mix can be stored as aliquots at −20°C for up to 1 year. Avoid freeze/thaw cycles if possible.

**Table:** NGS PCR Primer Mix (For step 48)

Reagent	Final concentration	Amount
NGS_PCR1 Primer (50 μM)	25 μM	50 μL
NGS_PCR2 Primer (50 μM)	25 μM	50 μL
**Total**	**N/A**	**100 μL**

Primer mix can be stored as aliquots at −20°C for up to 1 year. Avoid freeze/thaw cycles if possible.

***Alternatives:*** Agencourt Ampure XP beads (Beckman Coulter, Cat#A63880) are an alternative to the Sera-mag beads for the DNA clean-up steps. Although more expensive, Agencourt Ampure XP beads are ready to use, while Sera-mag beads need to be diluted in PEG buffer (see above) prior to use.

Other types of equipment that are required but do not have to be from a specific manufacturer (Equipment used will be listed in the key resources table (KRT)):

• Benchtop centrifuge with spinning capability at 20,000 *g*.
• DNA analyzer for size and quality control of samples.
• DNA fluorometer.
• Magnetic rack (96-well or one that can fit 0.2 mL PCR tubes).
• PCR machine.
• Pestle and electric drill (depending on tissue type).
• Sonicator.
• Temperature controlled metal heat block (up to 95°C).

## Step-by-step method details

### Extraction of genomic DNA

**Timing: 1 h; enzyme incubation times variable (1 h–16 h)**

The aim of this step is to extract and isolate genomic DNA from the samples, using the QIAamp DNA Micro Kit. Depending on the type of tissue or amount of material being processed, there are two methods for initial tissue homogenization.

1. Pre-heat a heat block to 56°C.
2. Initial tissue homogenization via one of two options:
  a. AL Buffer Protocol: recommended for whole Drosophila embryos, whole larvae, whole adult Drosophila heads (with additional mechanical homogenization) and tissue culture cells (no additional mechanical homogenization).
    i. Take the samples (stored without buffer) from −80°C and add 180 μL 1×PBS to the Eppendorf tubes.
      ***Optional:*** For tissue containing gut or any tissue with high concentrations of nucleases and/or proteases, add 145 μL of 1×PBS and 40 μL 500 mM EDTA (50 mM final concentration) to the Eppendorf tube instead.
    ii. Add 20 μL RNase (12.5 μg/mL stock solution) and gently mix.
      ***Optional:*** If samples (whole embryos, whole larvae, adult heads) require mechanical homogenization, use a sterilized pestle (washed in 100% ethanol) attached to an electric drill.
    iii. Add 20 μL Proteinase K (from QIAamp DNA Micro Kit), mix gently (by pipetting up and down or flicking the tube gently) then leave for 1 min at 21°C–24°C.
    iv. Add 200 μL Buffer AL, gently invert-mix roughly 50 times and incubate at 56°C for 10 min or 16 h, until the sample is completely lysed and digested.
      **CRITICAL:** Make sure the sample is completely digested.
    v. Cool to 21°C–24°C, add 200 μL 100% ethanol and mix by gently inverting.
  b. ATL Buffer protocol: Recommended for small volumes (<10 μL) of dissected tissue or cut larvae.
    i. Add 20 μL of 500 mM EDTA (50 mM final concentration and 20 μL of Proteinase K to 180 μL of ATL Buffer, mix by vortexing.
    ii. Take the samples (stored without buffer) from −80°C and add mixture from the previous step. Mix gently by inverting the tube.
    iii. Incubate at 56°C until completely digested and gently invert the tube occasionally to mix.
      ***Note:*** Depending on the type of tissue, digestion times can take from 1 h–16 h.
      ***Optional:*** If the sample is not properly digested after an 16 h incubation, add another 180 μL Buffer ATL + 20 μL Proteinase K to the sample and incubate for several more hours. Note that the volumes of RNase, Buffer AL and ethanol added in the subsequent steps will be doubled.
    iv. Add 20 μL RNase (12.5 μg/mL stock solution), mix by inverting the tube and incubate at 21°C–24°C for 2 min.
    v. In a separate tube, mix 200 μL buffer AL and 200 μL 100% ethanol by vortexing. (Total volume of 400 μL needed per sample).
    vi. Add 400 μL of Buffer AL/100% ethanol mix to each sample, mix well by gently inverting and flicking the tubes.
      ***Note:*** If a precipitate develops during this step, reheat the sample to 50°C–56°C for 1 min before mixing.
3. Add all of the solution from either steps 2a.(v.) or 2b.(vi.) to a spin column (QIAamp DNA Micro Kit).
4. Spin (>6,000 g) at 21°C–24°C for 1 min; discard the flow-through and collecting tube.
5. Add 500 μL AW1 solution and spin (>6,000 g) for 1 min; discard flow-through and collecting tube.
6. Add 500 μL AW2 solution and spin (>6,000 g) for 1 min; discard flow-through and collecting tube.
7. Transfer the column to a new tube and spin at 20,000 g for 3 min to dry the column.

***Note:*** Spinning at maximum speed of a standard benchtop centrifuge is recommended. A Qiavac vacuum can be used for steps 3–6 if there are many samples to be processed, but the drying step must be done using a centrifuge.

8. Transfer the column to a new 1.5 mL Eppendorf tube, add 50 μL of buffer AE and leave at 21°C–24°C for a minimum of 10 min. Spin at (>6,000 g) for 1 min and **keep** the flow-through (elution).
9. Run 1 μL of the elution on a 0.8% agarose gel to check sample quality. The genomic DNA should be a single band on the top the gel and not a smear, which could indicate DNA shearing. Troubleshooting 1 and Troubleshooting 2.

***Optional:*** Step 9 can be done while proceeding with the next major step. Set aside 1 μL of elution for quality checking and use the remainder for the next step.

### Isolation of methylated DNA

**Timing: 1–2 days**

At this stage, the sample will be digested with DpnI, which only cuts at adenine-methylated GATC sites. After enzyme digestion, the genomic DNA will be cleaned up using the QIAGEN PCR purification kit. The DNA will also be ligated with adaptors as the methylated fragments will serve as a template for PCR amplification. To ensure that only methylated regions of the DNA are amplified, the sample is digested with DpnII, which cuts at unmethylated GATC fragments.

10. Transfer 43.5 μL of elution to a new 1.5 mL Eppendorf tube.
11. In a separate tube, prepare a master mix with 5 μL of NEB CutSmart buffer and 1.5 μL of DpnI enzyme per sample, flick to mix and spin down.
12. Add 6.5 μL of the mix from above to the elution, very gently flicking the tube or pipetting up and down with a P1000 pipette to mix. Digest the mixture for 2 h–16 h at 37°C.

**CRITICAL:** Do not vortex this mixture as this can lead to shearing of genomic DNA.

13. Clean up the digested DNA according to the instructions in the QIAGEN PCR purification kit.
  a. Elute in a final volume of 32 μL of dH_2_O. Pipette the H_2_O directly on to the filter in the spin column and leave for 5 min at 21°C–24°C before spinning.

**Pause point:** DNA can be stored for up to 6 months at −20°C.

14. Measure the DNA concentration using a Qubit or NanoDrop fluorometer. Dilute samples to a maximum of 750 ng in 15 μL of dH_2_O. If the amount of DNA is lower than 750 ng, use 15 μL of the undiluted elution.

***Note:*** It is not unusual to have very low yields of DNA (minimum 5 ng) at this stage, as uncut genomic DNA (which should compose the majority of the sample initially) will be discarded during the purification steps. The yield of DNA is dependent upon the starting material, cell type and number of cells that are profiled by NanoDam.

***Optional:*** The diluted elution or any unused DNA can be stored at −20°C (as spare sample or for troubleshooting if necessary).

15. Transfer 15 μL of sample to 0.2 mL PCR tubes.
16. Add 4 μL of pre-made adaptor ligation buffer and 1 μL of T4 DNA ligase to the sample, mixing gently. Adaptor ligation buffer and T4 DNA ligase can be premixed in a master mix.
17. Using a PCR machine, incubate the ligation reaction for 2 h at 16°C, then 10 min at 65°C to inactivate the T4 DNA ligase.
18. Add 19 μL of pre-made DpnII digestion buffer and 1 μL of DpnII enzyme. These two can be premixed in a master mix before adding to the sample.
19. Digest at 37°C for a minimum of 2 h and maximum of 16 h (overnight).
20. Heat inactivate the enzyme by incubating the mixture at 65°C for 20 min.

### Further purification of methylated DNA

**Timing: 40 min**

This cleaning step greatly improves the efficiency of NanoDam-PCR through the removal of the buffer solution from previous steps. This step is recommended but optional if there are time constraints to the experiment. Clean-up is done using Sera-mag beads.

21. Add 60 μL of Sera-mag beads to the 40 μL sample (bead volume=1.5 × sample volume) and mix well by vortexing.
  a. Vortex for around 5 s and pulse down for 5 s. Rapid mixing of beads and sample is important.
22. Incubate at 21°C–24°C for 10 min.
23. Place on magnetic stand for 2 min or until the solution is clear.
24. Discard the supernatant.
25. Wash for 30 s with 190 μL 80% ethanol/H_2_O.
26. Let tubes stand for 1 min to air dry (avoid over-drying).
  a. A P10 pipette can be used to remove the last drops of 80% ethanol.
27. Resuspend in 32 μL of dH_2_O and remove from the magnetic stand.
28. Mix well and incubate for 2 min at 21°C–24°C.
29. Place on magnetic stand for 2 min or until the solution is clear.
30. Put 30 μL of the supernatant into a new clean 0.2 mL PCR tube.

### NanoDam-PCR: Amplification of methylated DNA fragments

**Timing: 2.5 h**

Using the digested methylated DNA fragments as a template, fragments can be amplified via PCR.

31. Add 19 μL of MyTaq Master Mix and 1 μL MyTaq HS DNA polymerase to the supernatant from step 35 and mix well. MyTaq Master Mix and MyTaq HS DNA polymerase can be premixed in a master mix.
32. Run PCR with the following conditions (total volume 50 μL):

**Table:** PCR cycling conditions

Steps	Temperature	Time	Cycles
1° Extension	72°C	10 min	1
1° Denaturation	95°C	30 s	1
1° Annealing	65°C	5 min	1
1° Extension	72°C	15 min	1
2° Denaturation	95°C	30 s	3 cycles
2° Annealing	65°C	1 min	
2° Extension	72°C	10 min	
3° Denaturation	95°C	30 s	17 cycles
3° Annealing	65°C	1 min	
3° Extension	72°C	2 min	
Final extension	72°C	5 min	1
Hold	4°C	Forever

**Pause point:** PCR-amplified DNA can be stored 16 h at 4°C or for up to 6 months at −20°C.

### Sonication and quality checks

**Timing: 16 h**

The DNA from the previous step is purified and the adaptors used for PCR amplification are removed. The quality checks are performed prior to the preparation of the libraries for next-generation sequencing.

33. Transfer the 50 μL sample to a 1.5 mL Eppendorf tube and purify the DNA following the instructions of the QIAGEN PCR purification kit. Elute in 32 μL of dH_2_O, leaving it for 5 min before the final spin.
34. Run 1 μL of the elution on a 0.8% agarose gel for a quality check. A smear between 400 bp–2 kb is expected.
35. Measure DNA concentration using Quantus/Qubit/NanoDrop. Troubleshooting 3.
36. Dilute samples to 2 μg DNA (or less) in 90 μL of dH_2_O in 1.5 mL or 0.2 mL sonication tube.

**CRITICAL:** Using tubes designed for sonication is important for consistent results.

37. Add 10 μL of NEB CutSmart buffer and mix well, cool on ice.
38. Sonicate the DNA to generate an average fragment size of roughly 300 bp.
  a. If using a Diagenode Bioruptor/Bioruptor Pico (at 4°C), switch on cooling unit at least 10 min before use, until the water temperature is 4°C.
  b. Sonicate for 6 cycles (30 s on, 20 s off) on high power if using Diagenode Bioruptor with 1.5 mL tubes or 17 cycles (30 s on, 20 s off) if using Diagenode Bioruptor Pico with 0.2 mL tubes.

**CRITICAL:** The sonication conditions depend on the type of sonicator used and should be optimized appropriately. Slight variations of fragment size are acceptable but very large variations (>600 bp) may impede clustering efficiency on the sequencing flow cell and thus the sequencing yields.

***Optional:*** Check the fragment size on the Tapestation (genomic tape) to ensure that the average fragment size is around 300 bp. If the sonication conditions have been optimized, it is not necessary to check fragment size for every experiment. Troubleshooting 4.

39. Add 1 μL AlwI enzyme, mix well and digest 2 h–16 h at 37°C.

***Note:*** AlwI digestion (which removes the dsAdR primer) can be carried out either before or after sonication. AlwI cannot be heat inactivated but will not affect the steps downstream.

40. Transfer 70 μL of each sample to 8-well PCR strips for library preparation.

**Pause point:** Sonicated DNA can be stored for up to 6 months at −20°C.

### Sequencing library preparation

**Timing: 3–4 h**

This step involves the generation of libraries for sequencing and can be summarized as follows: purification of DNA, adjusting concentration, end repair, adenylation of 3′ ends, sequencing adaptor ligation, another round of DNA purification, enrichment of DNA fragments and a final DNA clean-up.

41. Perform DNA cleanup via bead purification.
  a. Add 105 μL Sera-mag beads to 70 μL sample and mix well.
    i. Vortex and pulse down for 5 s. Rapid mixing of beads and sample is important.
  b. Incubate at 21°C–24°C for 10 min.
  c. Place on magnetic stand for 2 min or until the solution is clear.
  d. Discard the supernatant.
  e. Wash for 30 s with 190 μL 80% ethanol/dH_2_O. Perform 2 washes.
  f. Let samples stand for 1 min to air dry (avoid over-drying).
    i. A P10 pipette can be used to remove the last drops of 80% ethanol.
  g. Resuspend in 25 μL Resuspension buffer and remove from the magnetic stand.
  h. Mix well by vortexing and incubate for 2 min at 21°C–24°C.
  i. Place on magnetic stand for 2 min or until the solution is clear.
  j. Put 22.5 μL of the supernatant into a new, clean PCR tube.

**Pause point:** DNA can be stored for up to 6 months at −20°C.

42. Measure DNA library concentration by using 1 μL on the Qubit or similar fluorometer.
43. Dilute samples to (no more than) 500 ng of DNA in 20 μL Resuspension buffer.
44. End repair of the DNA fragments by:
  a. Add 7.5 μL End Repair Buffer to the diluted samples.
  b. Add 2.5 μL End Repair Enzymes and mix well by vortexing (total volume at this stage: 30 μL). End Repair Buffer and End Repair Enzymes can be premixed in a master mix.
  c. Incubate for 30 min at 30°C.
  d. Heat inactivate enzymes for 20 min at 75°C.
45. Adenylate 3′ ends by:
  a. Add 0.75 μL Klenow 3′ to 5′ exo-enzyme and mix well.
  b. Incubate for 30 min at 37°C.
46. Proceed immediately to adaptor ligation:
  a. Add 2.5 μL of the relevant adaptor.
    **CRITICAL:** Try to limit barcode base overlap (Table 1) as much as possible, especially if multiplexing 4 libraries or fewer. If barcodes are too similar, this may reduce the number of reads passing the filter. Refer to Illumina recommendations for more information.
  b. Add 2.5 μL of NEB Quick Ligase enzyme.
  c. Incubate for 10 min at 30°C (Total volume at this stage: 35 μL).
  d. Add 5 μL of 0.5 M EDTA to stop the ligation.
    **Pause point:** DNA can be stored for up to 6 months at −20°C.
47. Perform bead clean-up of DNA:
  a. Add 40 μL Sera-mag beads to the 40 μL sample and mix well.
    i. Vortex and pulse down for 5 s. Rapid mixing of beads and sample is important.
  b. Incubate for 10 min at 21°C–24°C.
  c. Place on magnetic stand for 2 min or until the solution is clear.
  d. Discard the supernatant.
  e. Wash for 30 s with 190 μL 80% ethanol/dH_2_O. Perform 2 washes.
  f. Let samples stand for 1 min to air dry (avoid over-drying).
    i. A P10 pipette can be used to remove the last drops of 80% ethanol.
  g. Resuspend in 22.5 μL Resuspension buffer and remove from the magnetic stand.
  h. Mix well by vortexing and incubate for 2 min at 21°C–24°C.
  i. Place on magnetic stand for 2 min or until the solution is clear.
  j. Put 20 μL of the supernatant into a new, clean PCR tube.

**Pause point:** DNA can be stored for up to 6 months at −20°C.

48. Enrich the DNA fragments by:
  a. Add 5 μL NGS PCR Primer mix.
  b. Add 25 μL NEBNext High-Fidelity 2× PCR Master Mix. NGS PCR Primer mix and NEBNext High-Fidelity 2× PCR Master Mix can be premixed in a master mix.
  c. Perform PCR with the following conditions (total volume: 50 μL):

**Table:** PCR cycling conditions

Steps	Temperature	Time	Cycles
Denaturation	98°C	30 s	1
Denaturation	98°C	10 s	6–8 cycles
Annealing	60°C	30 s	
Extension	72°C	30 s	
Final extension	72°C	5 min	1
Hold	4°C	forever

49. Perform a final round of bead clean-up of the sample:
  a. Add 45 μL Sera-mag beads (0.9× sample volume) to the 50 μL sample and mix well.
    i. Vortex and pulse down for 5 s. Rapid mixing of beads and sample is important.
  b. Incubate for 10 min at 21°C–24°C.
  c. Place on magnetic stand for 2 min or until the solution is clear.
  d. Discard the supernatant.
  e. Wash for 30 s with 190 μL 80% ethanol/dH_2_O. Perform 2 washes.
  f. Let samples stand for 1 min to air dry (avoid over-drying).
    i. A P10 pipette can be used to remove the last drops of 80% ethanol.
  g. Resuspend in 32.5 μL Resuspension buffer and remove from the magnetic stand.
  h. Mix well by vortexing and incubate for 2 min at 21°C–24°C.
  i. Place on magnetic stand for 2 min or until the solution is clear.
  j. Put 30 μL of the supernatant into a new, clean PCR tube.

### Library quality check, multiplexing and sequencing

**Timing: 1 h (QC + Multiplexing)**

A final quality check is performed prior to multiplexing all the libraries generated. Samples are then sequenced on an Illumina sequencer.

50. Check the DNA sample generated from step 49 on an Agilent Tapestation or Bioanalyzer. Take note of the average fragment length for each sample for step 52.
51. Measure DNA concentration with a Qubit or Quantus fluorometer.

Troubleshooting 5Troubleshooting 6Troubleshooting 7.

***Note:*** If there is 500 ng of starting material, the final concentration should be 15–30 ng/μL.

***Optional:*** Libraries can be more accurately quantified using the NEBNext® Library Quant Kit for Illumina.

52. Calculate the molarity of each sample using fragment length and concentration and pool samples to give a final DNA concentration of 20 nM (ensure that all libraries are at equal concentration).
  a. Molarity = (1500/Average Fragment Size) × (DNA concentration in ng/μL).
  b. Library volume to add = (Final DNA concentration (20 nM)/Molarity) × (Final volume (50 μL)/Number of samples).
  c. H_2_O to add = Final volume (50 μL) – Sum of library volumes.

***Note:*** The final DNA concentration of 20 nM was determined based on the recommended concentration for Illumina sequencing.

53. Check the multiplexed library on the Agilent Tapestation before proceeding to sequencing.
54. Sequence single-ended 50 nt (SE50) or single-ended 100 nt (SE100) reads on an Illumina sequencer. Troubleshooting 8.

**CRITICAL:** For *Drosophila* samples, aim for a minimum of 20 million reads per library. Other organisms may require a higher number of reads, depending on genome size. For instance, around 40 million reads for human samples.

### Computational analysis and visualization of NanoDam data

**Figure 3:**
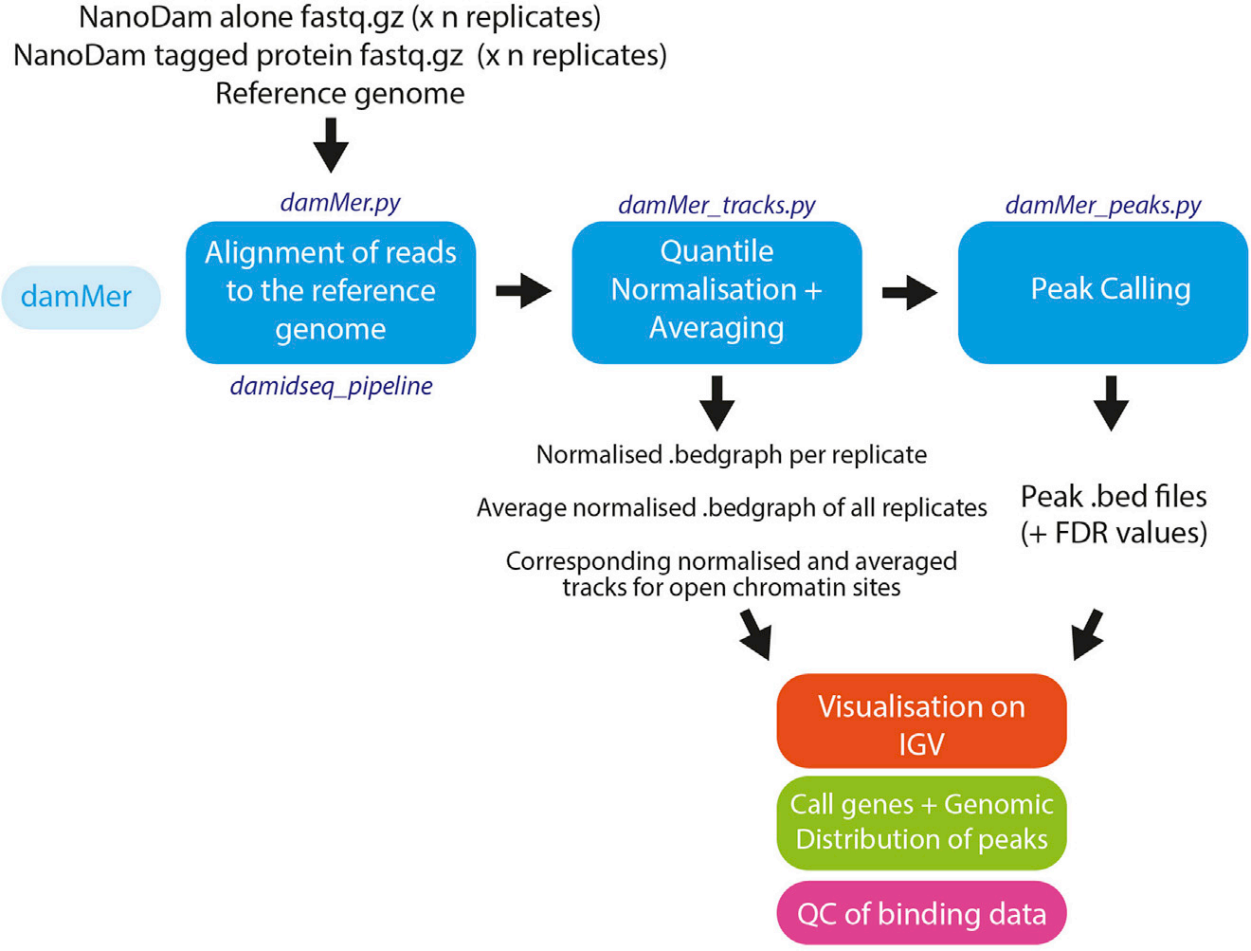
Summary flowchart of the basic workflow of NanoDam binding data analysis The *damMer* suite consists of 3 python scripts that build on each other and is used to run all pairwise comparisons between the experimental and control samples. The resulting track and peak files can be used in downstream analyses.

The workflow for analyzing NanoDam data builds upon the existing *damidseq_pipeline* (Marshall and Brand, 2015) and takes into account cross-comparisons of multiple replicates. It is composed of a suite of Python scripts (collectively called *damMer*) which generates normalized binding tracks (∗.bedgraph format) and identifies statistically significant and reproducible peaks (across replicates) (∗.bed format). Data analyzed by this pipeline can then be used for other downstream analysis, such as ChIPseeker, principal component analysis and can be visualized using the Integrative Genome Viewer (IGV) (Robinson et al., 2011) (Figure 3).

Here we will summarize the main steps of using this pipeline for a NanoDam experiment to generate binding tracks and peak sets. Additional steps on assessing the quality of binding data, including comparisons of replicates and complexities of libraries generated from the experiments will also be discussed. Please note that additional technical notes to supplement the NanoDam data analysis are provided in the corresponding GitHub repository: https://github.com/AHBrand-Lab/NanoDam_analysis. All references to Python scripts (i.e., ∗.py) and R markdowns (i.e., ∗.Rmd) refer to code that was deposited in this repository.

*damMer* applies the statistical framework of the *damidseq_pipeline* in an automated manner to all possible pairs of the provided NanoDam-tagged protein and NanoDam-alone samples, averaging across all pairs. As these individual tasks benefit greatly from parallelization, the suite utilizes the workload manager slurm (https://slurm.schedmd.com/documentation.html). All tasks (e.g., copying fastq files, running *damidseq_pipeline*, averaging, quantile normalization) are submitted as individual jobs to slurm, which automatically schedules these jobs.

55. Install or download the following packages and scripts in preparation for running damMer:
  a. To run *damMer.py*:
    i. samtools.
    ii. bowtie2.
    iii. damidseq_pipeline.
  b. To run damMer_tracks.py:
    i. bedGraphtoBigWig.
    ii. quantile_norm_bedgraph.pl.
    iii. average_tracks.pl.
    iv. MACS2.
56. Perform “quality check” on NanoDam sequencing files (∗.fastq format) by filtering out low quality reads, removing residual adapter sequences or trim nucleotides not passing Phred quality score thresholds.
  a. Use *cutadapt* (Martin, 2011) to trim adaptors (‘--action=trim’, default setting).
  b. Discard reads that are too long after adapter trimming (--maximum-length=50) or too short (--minimum-length=30).
  c. Monitor results of filtering using *fastqc* and use *multiqc* to aggregate results across all sequencing files to facilitate comparison.

>files=($(find . -type f -iname “∗.fastq∗”))

>for f in ${fs[@]}; do ∖

> fastqc ${f}; ∖

> cutadapt -m 30 -M 50 -o ${f/fastq/_trim.fastq} ${f}; ∖

> fastqc ${f/fastq/_trim.fastq}; ∖

>done

>multiqc

**Figure 4:**
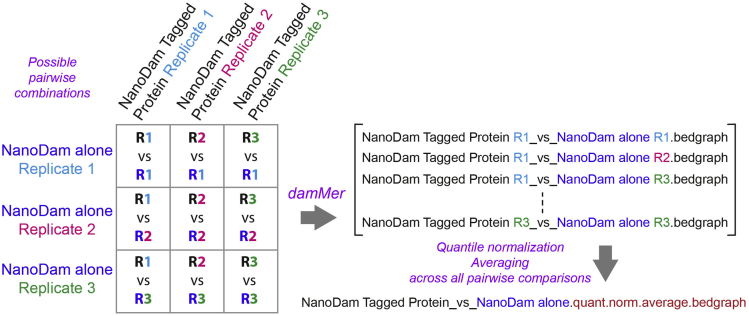
Diagram showing the naming convention employed in the *damMer* suite and the pairwise manner in which all samples are compared to each other All names include the ∗_vs_∗ phrase to separate the indicators for the experimental and control samples.

***Note:*** Other visualization methods can be used (e.g., UCSC genome browser) though IGV is the standard.

57. Run the damMer suite of Python3 scripts in the following order: (1) *damMer.py*, (2) *damMer_tracks.py*, (3) *damMer_peaks.py.*
  a. *damMer.py*: maps trimmed reads to a reference genome via the *damid_seq pipeline.*
    i. Obtain the relevant reference genome for the experiment (e.g., from https://ftp.ncbi.nih.gov/genomes/refseq/ or Illumina iGenomes https://support.illumina.com/sequencing/sequencing_software/igenome.html).
    ii. Prepare a file with bowtie-2 indices for the corresponding genome (acquired online: http://bowtie-bio.sourceforge.net/bowtie2/index.shtml or generated with the bowtie2-build command based on the genome sequence in ∗.fasta format).
    iii. Prepare a file specifying all genomic NanoDam-methylation sites (i.e., GATC-motifs) which can be generated by the *gatc.track.maker.pl* script (∗.gff format).
    iv. To avoid leaving samples out, provide all NanoDam-tagged protein and NanoDam-alone sequence files as shell arrays.
      >ctrls=($(find . -type f -iname “∗_trim.fastq∗” -and -iname “dam_∗”))
      >exps=($(find . -type f -iname “∗_trim.fastq∗” -and -iname “tf_gfp_∗”))
      >python3 damMer.py -e ${exps[@]} -c ${dam[@]} ∖
      >  -i path/to/bowtie2_index ∖
      >  -g path/to/motifs.GATC.gff ∖
      >  -b path/to/bowtie2 ∖
      >  -s path/to/samtools ∖
      >  -q path/to/damidseq_pipeline_vR.1.pl
      ***Note:****damMer.py* will generate folders for every pairwise comparison (i.e., ∗_vs_∗, Figure 4), copy the required fastq files with trimmed reads into them, validate the file formats, generate shell scripts to run the *damidseq_pipeline* in all folders and submits them as jobs to slurm while ensuring that all jobs are running. A separate log-file with all information provided to and by *damMer.py* is also generated which enables users to retrace all parameters and arguments used to run this script. Similarly, all shell scripts submitted to slurm are kept.
      **CRITICAL:***damMer_tracks.py* will adjust names for all files generated by *damMer.py*, therefore it is not recommended to manually change filenames in the folder created by *damMer.py*.
  b. *damMer_tracks.py*: generates tracks of averaged, normalized binding intensities for the NanoDam experiment across the entire genome using tracks generated across all pairwise comparisons of NanoDam-tagged protein and NanoDam-alone samples (Figure 4).
    i. To include all tracks generated by all pairwise comparisons by specifying the folders (i.e., ∗_vs_∗) in which they are located when running *damMer_tracks.py*.
    ii. Prepare a file with the corresponding genome sizes (∗.tsv format) (e.g., dm.chrom.sizes from https://www.encodeproject.org/files/dm6.chrom.sizes/).
      >dirs=($(find . -type f -iname “∗_vs_∗”))
      >python3 damMer_tracks.py -r ${dirs[@]} ∖
      >  -o ∗output_folder_name∗ ∖
      >  -p ∗name_NanoDam_fusion_protein∗ ∖
      >  -c ∗name_NanoDam∗ ∖
      >  -m path/to/MACS2 ∖
      >  -n path/to/quantile_norm_bedgraph.pl ∖
      >  -a path/to/average_tracks.pl ∖
      >  -b path/to/bedGraphToBigWig ∖
      >  -l path/to/∗genome∗.chrom.sizes
      ***Note:****damMer_tracks.py* creates a folder ending in ∗_tracks (beginning of folder name defined via ‘-o’ argument) that includes copies of all individual tracks from all folders generated by *damMer.py* (i.e., ∗_vs_∗), as well as quantile normalized and averaged versions in ∗.bedgraph and ∗.bw (bigwig) format.
      ***Optional:*** Alternatively, all individual tracks can also be read into R via the functions *import.bedgraph()* or *import.bw()* into a combined matrix and quantile normalized via preprocessCore::normalize.quantiles() (see *genomewide_correlation.Rmd*).
      ***Note:****damMer_tracks.py* also submits jobs for peak calling with MACS2 based on bam-files acquired from *damMer.py* (i.e., ∗-ext300.bam). For each pairwise comparison, peaks will be called for the bam-file corresponding to the tagged protein of interest compared to its NanoDam-alone control. As NanoDam-dependent methylation signals are not as locally confined as ChIPseq-signal, we always obtain broad peaks (i.e., MAC2 argument --broad).
      To obtain both normalized tracks for the binding intensities of the tagged protein of interest and tracks specifying putative open chromatin sites, *damMer.py* makes use of *damidseq_pipeline_vR.1.pl* and *damMer_tracks.py* creates a second folder, named ∗_DamOnly_tracks. *damidseq_pipeline_vR.1.pl* is a modified version of the initial *damidseq_pipeline.pl* that also generates unnormalized (i.e., not compared to a control) NanoDam-alone tracks in line with the ideas of CATaDa (Aughey et al., 2018). Copies of these NanoDam-only tracks are gathered, quantile normalized to each other and averaged in the ∗_DamOnly_tracks folder by *damMer_tracks.py*.
  c. *damMer_peaks.py*: identifies sets of statistically significant peaks and sets of reproducible peaks for a defined list of FDR-thresholds (i.e., reproducible peaks that occur in at least 50% of all pairwise comparisons).
    i. This script will use the folder names (and file names) generated by damMer.py (i.e., ∗_vs_∗) and create “∗_peaks and ∗_DamOnly_peaks folders to store the final output peak files (code chunk 4).
      >dirs=($(find . -type f -iname “∗_vs_∗”))
      >python3 damMer_peaks.py -r ${dirs[@]} ∖
      >  -o ∗output_folder_name∗
      ***Note:*** All ∗.broadPeak files with peaks identified in individual pairwise comparisons will be gathered in a new ∗_peaks folder by *damMer_peaks.py*. The peaks in this set of files will be thresholded multiple times according to a defined list of 41 FDR cut-offs (i.e., -log_10_(FDR) = 0, 1, 2 ..., 5, 10 ... 100, 125 ..., 1900, 2000). All peaks left after thresholding with a particular FDR-value (-log_10_(FDR_peak_) ≥ FDR_threshold_) will be combined into a single file, sorted and merged to obtain a consensus set of peaks for each FDR, leaving the user with a set of 41 files corresponding to the FDR-values (i.e., ∗.mergePeak file format). In addition, all peaks are filtered by their appearance throughout the set of ∗.broadPeak files belonging to the pairwise comparisons and only peaks occurring in at least 50% across all files will be kept as reproducible set (i.e., ∗.reproPeak file format).
58. Download the latest version of Integrative Genome Viewer (IGV) and load the desired binding tracks (files in ∗.bedgraph or ∗.bw format) and significant peaks (files in ∗.bed format corresponding to the desired FDR).

### Analysis of NanoDam binding data quality

While the necessary quality check of the sequencing libraries accounts for low quality reads and nucleotides, as well as adapter contamination, the sensitivity of the assay and reproducibility of the experiment have to be determined separately. In order to detect problems occurring during NanoDam-induction and -methylation, library preparation and sequencing that may impact the entire library, genome-wide correlation analysis, signal enrichment analysis, and assessing the complexity of all sequenced libraries is recommended.

59. Perform genome-wide correlation analysis on unnormalized (i.e., normalization against a NanoDam-alone control) sequencing libraries.
  a. Use the ∗-ext3000.bam files (generated by *damMer.py*) located in its individual output folders (one per pairwise comparison).
  b. Map and bin the reads via *bamCoverage* from the *deeptools* suite (Ramírez et al., 2016) (Code chunk 5).
  c. The expected outputs are ∗.bedgraph files which can be read into R and genome-wide correlation analysis can be executed by following the workflow of *genomewide_correlation.Rmd.*
60. Perform correlation analysis on the normalized data derived from pairwise comparisons of NanoDam-tagged protein and control NanoDam-alone samples.
  a. Use the normalized ∗.bedgraph files generated from *damMer.py* and *damMer_tracks.py*, stored in the ∗_tracks folder.
  b. Process these files following the workflow of *genomewide_correlation.Rmd.*

>files=($(find . -type f -iname “∗.fastq∗”))

>for f in ${fs[@]}; do ∖

> gunzip -c ${f} | bowtie2 ∖

>  -x path/to/bowtie2_index ∖

>  -U - ∖

>  -S ${f/%.fastq∗/.sam};

> samtools view ∖

>  -Sb ${f/%.fastq∗/.sam} ∖

>  > ${f/%.fastq∗/.unsort.bam};

> samtools sort ∖

>  -o ${f/%.fastq∗/.sort.bam} ∖

>  ${f/%.fastq∗/.unsort.bam};

> samtools index ${f/%.fastq∗/.sort.bam};

> bamCoverage ∖

>  --bam ${f/%.fastq∗/.sort.bam}" ∖

>  --outFileName ${f/%.fastq∗/.sort.bam}.bin500.ext150.bedgraph" ∖

>  --outFileFormat bedgraph ∖

>  --binSize 500 ∖

>  --extendReads 150;

>done

61. Assess the intrinsic complexity of NanoDam libraries via two analyses:
  a. Use the *preseq c-curve* software (Daley and Smith, 2013) to calculate the alignment complexity of libraries: reads with unique sequencing information are plotted as a function of all reads across a gradually increasing number of reads included in the library (i.e., sequencing depth).
    i. Use the c-curve function on aligned reads in ∗.bam file format (Code chunk 6) or using the ∗-ext300.bam files in the individual folders for pairwise comparisons.
    ii. Plot the results of the output ∗.txt files in R as outlined in *signal_enrichment.Rmd* (section [5.0]).
      >fs=($(find . -type f -iname “∗.sort.bam”))
      >for f in ${fs[@]}; do ∖
      > preseq c_curve ∖
      >  -output ${f}_preseq.txt ∖
      >  -bam ${f};
      >done
  b. Perform cumulative enrichment analysis (Diaz et al., 2012) and generate a fingerprint plot which determines how the signal from the NanoDam-tagged protein samples can be differentiated from the background read distribution in the NanoDam-alone control samples.
62. Assess whether signal (i.e., tracks) are enriched on binding sites deemed statistically significant (i.e., peaks) by following the workflow of *signal_enrichment.Rmd* in R:
  a. Use the normalized, averaged tracks from NanoDam-tagged protein experiments (from *damMer_tracks.py*) and significant, reproducible peaks (∗.reproPeak files from *damMer_peaks.py*).
  b. Extract the binding signal over the peaks using the *extract_matrix()* function and plot the results.

>dirs=($(find . -type f -iname “∗_vs_∗”))

>for f in ${dirs[@]}; do ∖

> a=$(echo ${f} | awk -F_vs_ '{print $1}' | awk -F_ '{print $NF}'); ∖

> b=$(echo ${f} | awk -F_vs_ '{print $2}' | awk -F_ '{print $NF}'); ∖

> if [[ ${a} -ne ${b} ]] then ∖

>  echo ${f};

>  fsf+=${f};

> fi;

>done

>python3 damMer_tracks.py -r ${fsf[@]} ∖

> -o ∗output_folder_name∗

***Note:*** For these analyses it is recommended to use other NanoDam-tagged protein datasets with the same FDR-threshold as a comparison and negative controls derived from comparing NanoDam-alone samples to each other (e.g., NanoDam-alone_2_vs_NanoDam-alone_1 …, NanoDam-alone_3_vs_NanoDam-alone_4). This requires *damMer* to run on NanoDam-alone samples as experimental (*-e* argument) and control samples (*-c* samples). When starting *damMer_tracks.py* and *damMer_peaks.py*, the folders (i.e., ∗_vs_∗) where the same NanoDam-alone sample is both experimental and control (e.g., NanoDam-alone_1_vs_NanoDam-alone_1) can be left out (Code chunk 7).

## Expected outcomes

**Figure 5:**
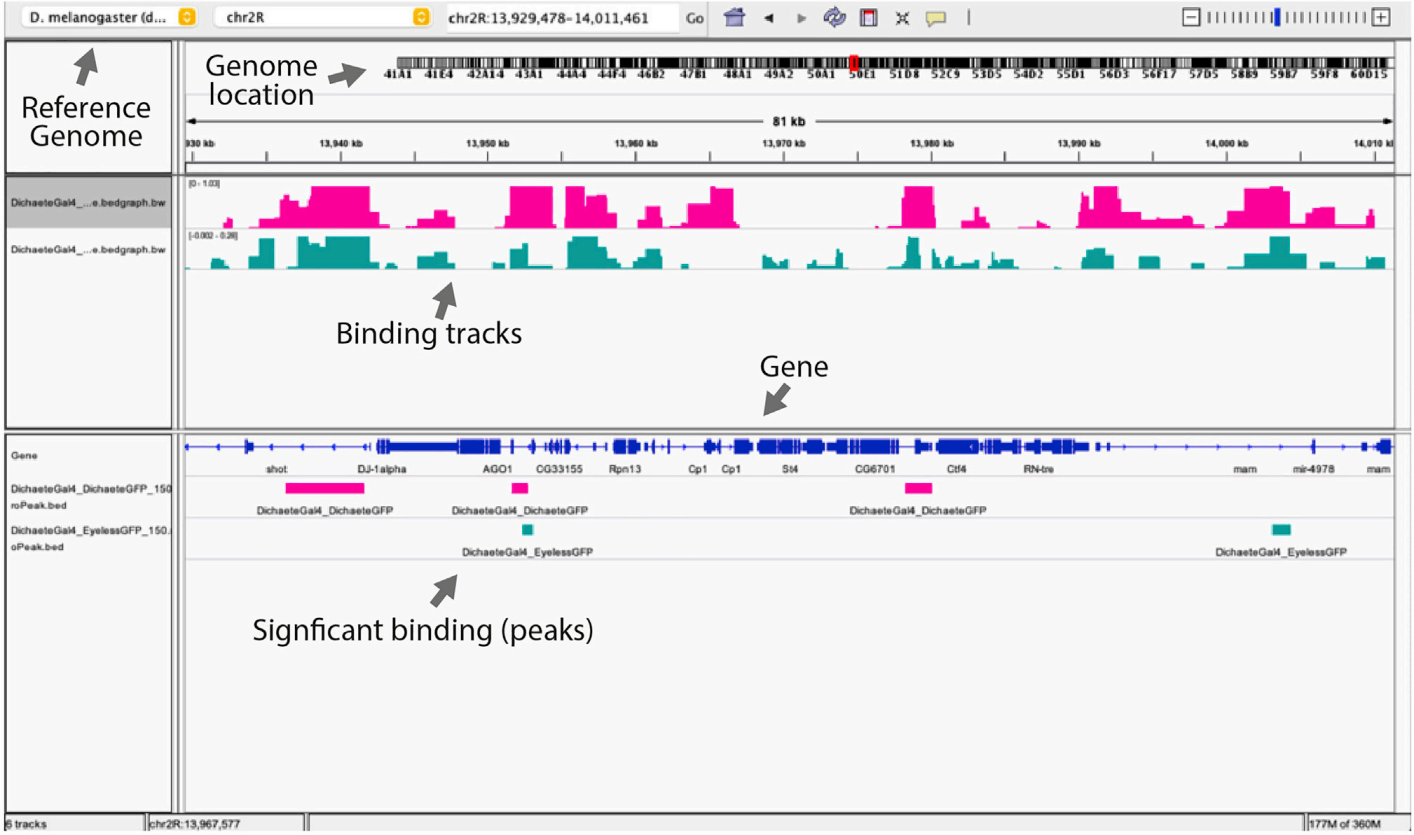
Diagram showing an example of visualized damMer output via IGV Tracks of two different NanoDam binding experiments are shown (pink and green) in the top half, while the bottom half displays the corresponding peaks (significant binding) in one particular genomic location.

By the end of the protocol, next generation sequencing data should be obtained from the NanoDam profiling experiment. The *damMer* python suite of scripts should generate genome-wide binding tracks of the NanoDam experiment in ∗.bedgraph format, normalized and averaged across all replicates and with all possible pairwise comparisons. Statistically significant binding can be determined by the identification of peaks, which are stored in ∗.bed format. Loading these files on IGV enables visualization of tagged protein binding across the whole genome (Figure 5).

Further downstream analysis can be performed after running damMer. The ChIPSeeker package (Yu et al., 2015) can be used to compare potential differential binding under different experimental conditions and to examine the binding distributions across the whole genome (preferential binding on promoters, exons, introns etc.).

### Quality check of NanoDam data (1): Genome-wide correlation of replicates

**Figure 6:**
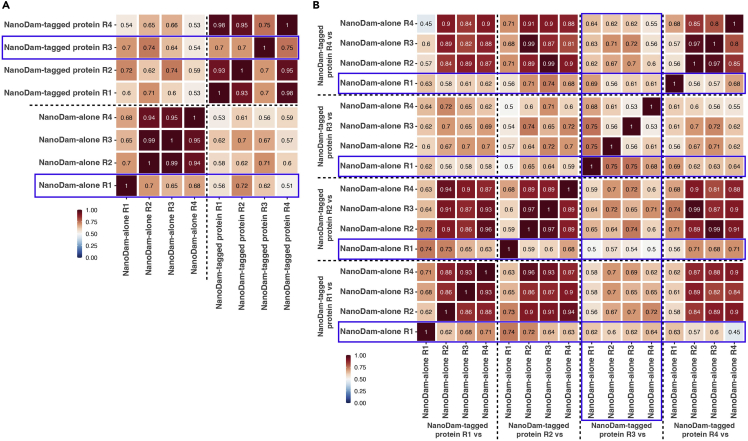
Quality check of the NanoDam-derived sequencing libraries (A) Correlation analysis of 4 NanoDam-alone and 4 NanoDam-tagged protein mock-samples showing the Pearson correlation coefficient for each pairwise comparison. Here, sample NanoDam-alone replicate 1 (R1) and NanoDam-tagged protein replicate 3 (R3) do not correlate sufficiently with the other respective samples (highlighted in blue). (B) When compared in a pairwise manner, these two samples (highlighted in blue) also reduce the Pearson correlation coefficients with other normalized, pairwise comparisons. In such a case, the two samples should be removed.

At the end of steps 59 and 60, Pearson correlation coefficients for the correlation of all samples against each other are calculated. It is recommended to keep individual libraries with a Pearson correlation coefficient of ≥0.9 compared to libraries of the same type (i.e., among NanoDam-tagged protein samples or NanoDam-alone samples) and pairwise comparisons with a Pearson correlation coefficient of ≥0.8 among the other comparisons of the same type (e.g., NanoDam-tagged protein_vs_NanoDam-alone comparisons derived from the same experimental setup (Figure 6).

### Quality check of NanoDam data (2): Complexity analysis

**Figure 7:**
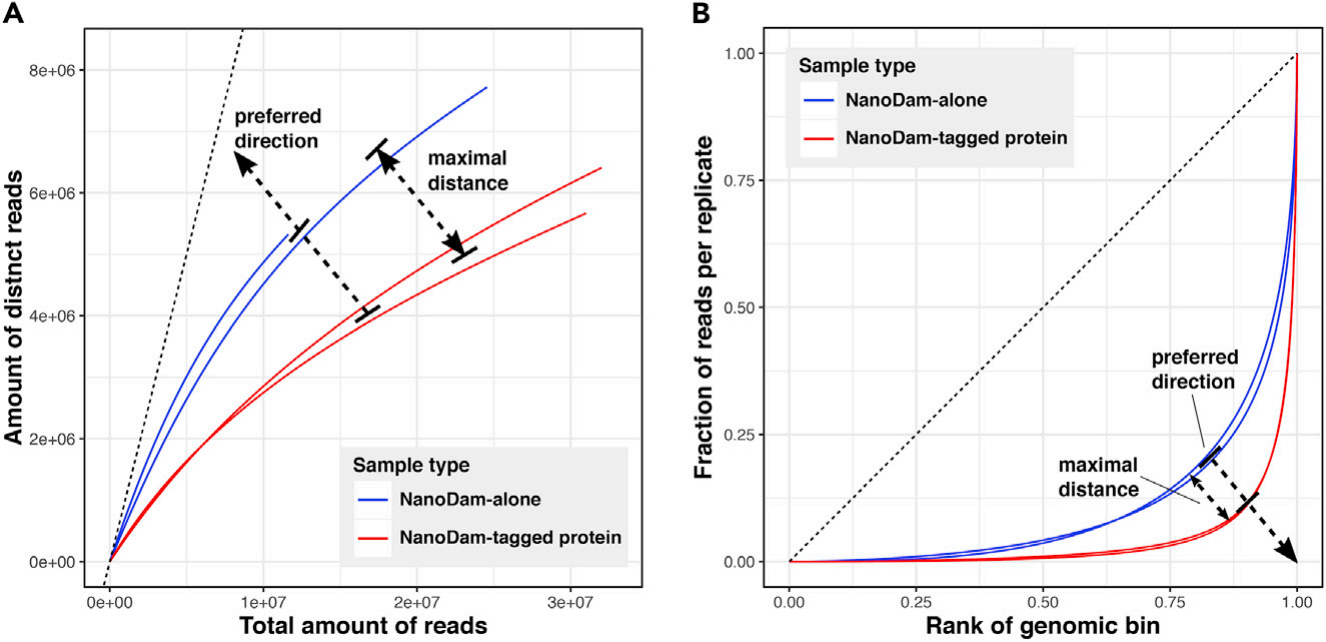
Diagrams showing the preferred profiles of NanoDam-derived sequencing libraries in alignment complexity (A) Preferred direction of curves in cumulative enrichment analysis. (B) Every curve represents either a NanoDam-alone or a NanoDam-tagged protein library. In both analyses, the further the NanoDam-tagged protein sample curves are away from the corresponding NanoDam-alone curves, the stronger is the specific signal enrichment at the actual binding sites of the NanoDam-tagged protein.

Libraries of NanoDam-alone and NanoDam-tagged protein samples should show the highest possible slopes in the complexity curves. At the same time, the curves for the two sample types should be as far apart from each other as possible with the NanoDam-tagged protein curves showing a lower slope as the NanoDam-alone curves. This indicates an enrichment of reads at binding sites of the tagged protein profiled by NanoDam (Figure 7A).

To further validate this enrichment and thus protein-binding signal across the genome, the fingerprint plots will elucidate an overall distribution of reads in a library across the genome. For this purpose, reads are quantified along the binned genome and the bins ranked according to the sum of reads falling into them. By plotting the cumulative fraction of reads along an increasing bin rank, the coverage of the genome by reads of the library in one curve can be evaluated. The closer the elbow of a library’s fingerprint curve is to the lower right corner of the plot, the more concentrated the library’s reads are in few bins. Similar to the complexity curves, both sample types, NanoDam-tagged protein and NanoDam-alone, should have the widest possible distance from one another (Figure 7B). This ensures a high signal enrichment (i.e., signal-to-noise-ratio). For both, complexity curves (step 61a) and fingerprint plots (step 61b.), an overlap of the curves for NanoDam-tagged protein- and NanoDam-alone-samples would mean an absence of specific binding sites.

### Quality check of NanoDam data (3): Signal enrichment analysis and selecting FDR values

Whether a NanoDam-experiment was successful can be examined by quantifying the enrichment of signal (i.e., tracks) on significant binding sites (i.e., peaks). The lower the number of reads mapping stochastically to the genome (i.e., noise, due to random DNA shearing during library preparation for example), and the higher the specificity of the reads to accumulate at binding sites (due to high affinity of the protein of interest for its binding motif), the higher the signal-to-noise ratio or signal enrichment. The closer the individual tracks from the pairwise comparisons are, the more reproducible the results are amongst individual libraries and experiments. In parallel, a high signal in the center of the peaks indicates a high signal enrichment. The curves for the negative controls should show an insignificant enrichment compared to the enrichment of the experimental pairwise comparisons. However, slight signal enrichment over peak centers is expected as this indicates stochastic methylation in open chromatin sites and presence of noise.

An analysis of signal enrichment on significant peaks analysis can also be used to determine which FDR-threshold (see *damMer_tracks.py* and *damMer_peaks.py*) should be used for defining the set of significant peaks. Dam- and by extension NanoDam-derived methylation can be separated into direct (primary) and indirect (secondary) methylation sites (Redolfi et al., 2019). Indirect or secondary methylation sites are open chromatin regions without bound NanoDam-tagged protein that are in close topological contact with the primary, direct sites bound by the tagged protein. High quality data will include both types of methylation sites. However, the more significant peaks are considered in signal enrichment analysis, the more secondary sites with a reduced signal enrichment will be included as well. A more stringent FDR-cut-off is recommended to focus on direct binding sites of the tagged protein of interest.

## Limitations

NanoDam mainly relies on the interaction of a GFP tagged protein with DNA, therefore it is important that the GFP tagging does not impair the function of the protein and specifically its interaction with DNA. When producing endogenously tagged cells or organisms, the function of the GFP tagged proteins should be assayed and alternative tagging strategies could be used to circumvent any problems: N-terminal tagging vs C-terminal tagging.

Another limitation of NanoDam is its resolution compared to ChIP-Seq. As in TaDa, it depends on the frequency of GATC sites in the genome (with median spacing of ∼190 bp in *Drosophila*).

The minimum number of cells required for a successful NanoDam experiment remains to be determined, nevertheless TaDa has been able to provide reliable data with as few as 10.000 cells, which could be extrapolated to NanoDam. The minimum proportion of cells expressing the protein of interest required in order to obtain proper NanoDam signal may depend on several factors, such as the binding affinity or potential cofactors of the protein of interest, the accessibility of target sites and nuclear concentration of the protein. Determining the optima for every tagged protein of interest is potentially feasible for small screens or targeted approaches, but impractical for assaying a multitude of factors across various conditions.

## Troubleshooting

### Problem 1

Genomic DNA shearing.

**Figure 8:**
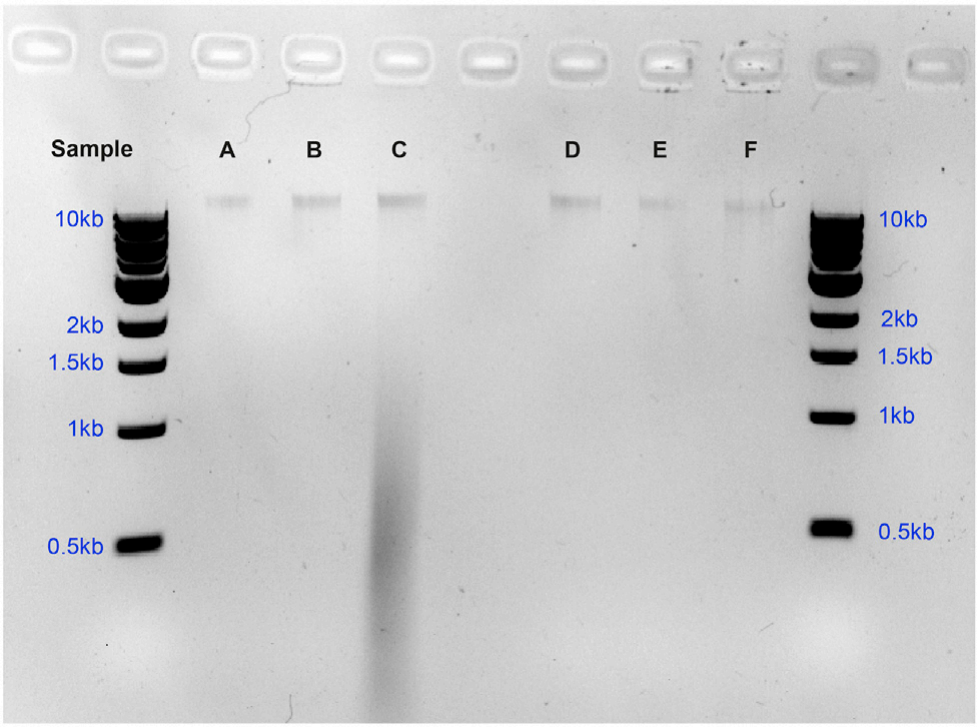
Gel showing quality of DNA after genome extraction 1 μL of individual sample preparations were run on lanes A–C and D–F. All lanes except lane C show good quality genome extractions, where a single high molecular weight band can be seen with very little to no smearing. Lane C shows smearing which may indicate DNA shearing or RNA contamination. In this situation, the sample from lane C should be discarded.

If a smear of DNA rather than a single high molecular weight band is observed in the agarose gel, it is likely that the DNA has been sheared during extraction and therefore the sample should be discarded: sheared DNA can ligate to the DamID adapter as if it had been methylated (Figure 8).

### Potential solution

Be gentler when extracting DNA, avoid vortexing and vigorous pipetting.

### Problem 2

Low amounts of genomic DNA.

If the DNA is barely visible it is likely that there are insufficient amounts of material.

### Potential solution

Use more starting material or more homogenization.

### Problem 3

Low PCR yield.

**Figure 9:**
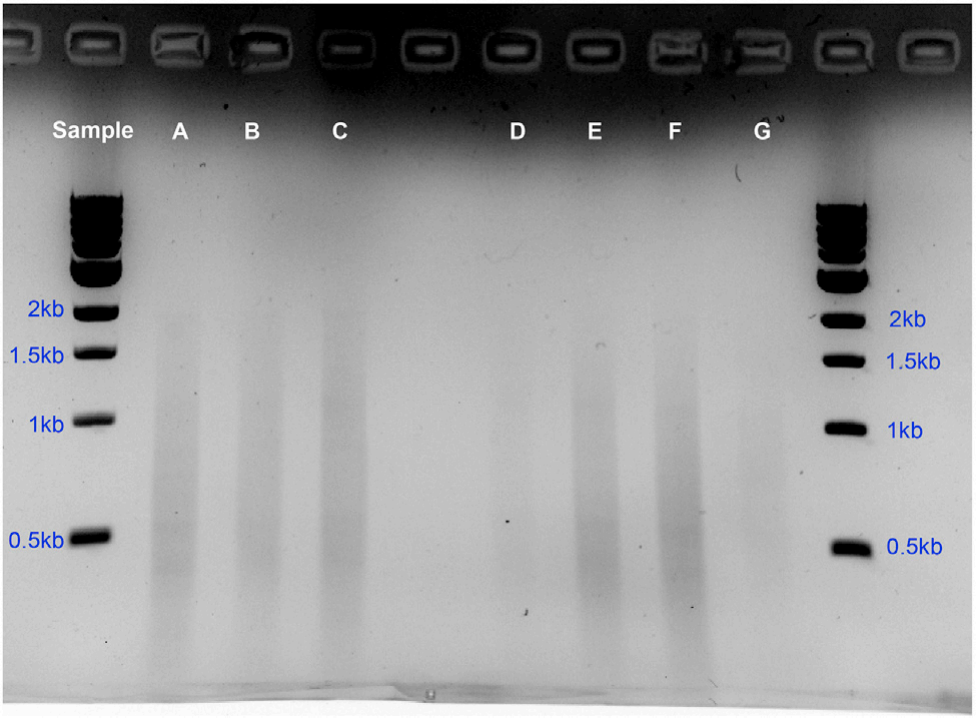
Gel showing individual samples after PCR amplification Lanes A–G also display smears indicative of successful amplification. Lanes D and G have lower concentrations of DNA but can still be used to produce sequencing libraries. Note the concentrations of DNA of the samples here range from 20–60 ng/μL (1 μL of DNA per sample to run gel).

If the DamID PCR yields low amounts of DNA it might be that the starting material was insufficient or the methylation signal is low (Figure 9).

### Potential solution

Use more starting material or change NanoDam induction time. Using more material or allowing NanoDam to methylate for longer periods may help. When increasing induction times, do it accordingly with your Dam only control.

### Problem 4

DNA fragment sizes bigger than expected.

**Figure 10:**
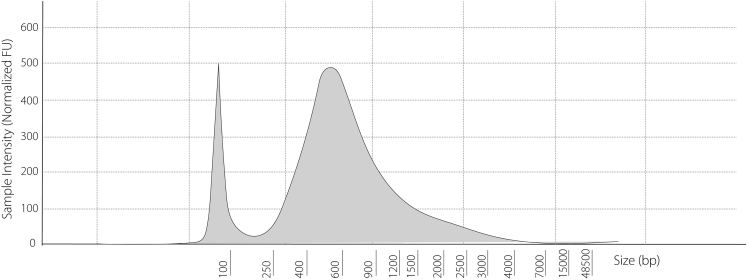
Tapestation plot showing successful sonication with optimal fragment distribution The majority of fragments range from 300–400 bp – if distribution is not in this range, sonication times will need to be optimized.

After sonication, a smear around 300–400 bp should be obtained. At this stage, we recommend using the Tapestation to visualize the distribution of fragments (see below) due to the relatively low concentrations of DNA. Any differences observed would suggest that the number of sonication cycles needs to be optimized (Figure 10).

### Potential solution

Increase number of sonication cycles.

### Problem 5

No DNA after preparation of sequencing library.

If all control points have been achieved up to this point, obtaining no DNA after library preparation would suggest that there has been a mistake in the preparation of the reactions.

### Potential solution

Repeat library preparation procedure with fresh reagents.

### Problem 6

Secondary peak in sequencing library.

**Figure 11:**
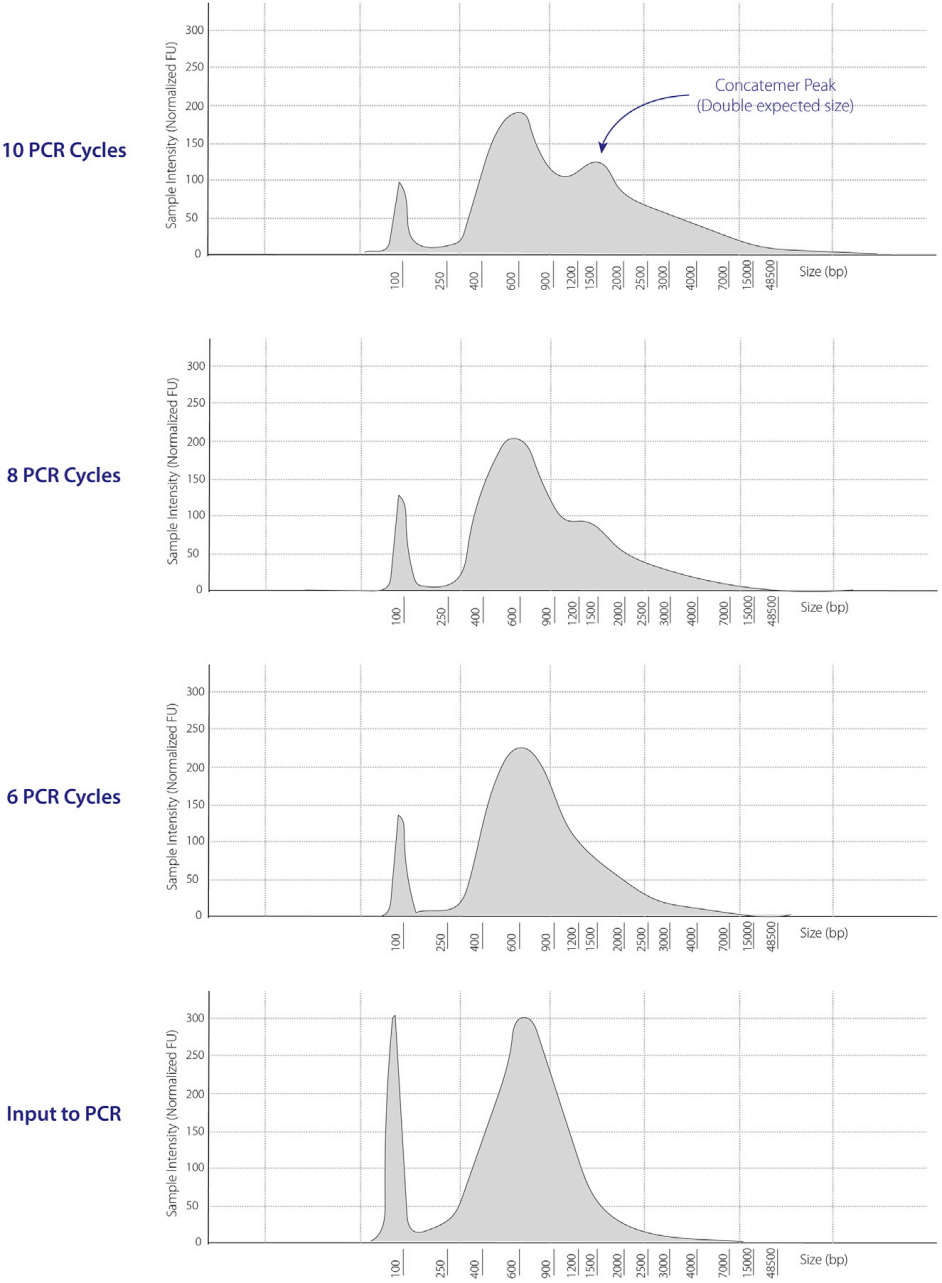
Tapestation plots showing concatemers when too many PCR cycles are used for amplification The relative concentrations (y-axis= sample intensity [FU]) of differently sized (x-axis= size [bp]) DNA fragments. 6 cycles of PCR are recommended as more cycles tend to produce secondary peaks (concatemers), which affects the quality of the sequencing library. The peak of smaller DNA fragments (at 100 bp) is indicative of adaptor dimers.

Probably due to exhaustion of the PCR resulting in concatemers (Figure 11).

### Potential solution

Reduce input DNA quantity or number of cycles. If this secondary peak is seen in the Tapestation genomic plots, reduce the number of PCR cycles. 6 is recommended, as this issue is generally seen when using 8 or more cycles.

### Problem 7

Library has adapter contamination.

If a peak around 120–130 bp is observed it would suggest the presence of contaminating adapter dimers, which would affect the sequencing yield.

### Potential solution

Repeat bed-cleanup step at with 0.9× volumes of beads.

### Problem 8

Low number of reads/low percentage of reads mapped to genome.

Adaptor dimers or adaptor concatemers might still be present in the sequencing library. Failure to remove initial DamID adaptors with AlwI could also be the cause. Contamination with foreign DNA.

### Potential solution

Use fresh AlwI enzyme and buffer. Make sure to keep pipettes and workspace clean and use filter tips when processing samples.

## Resource availability

### Lead contact

Further information and requests for resources and reagents should be directed to and will be fulfilled by the lead contact, Andrea Brand (a.brand@gurdon.cam.ac.uk).

### Materials availability

Plasmids and fly stocks generated in this study are available upon request.

## Data Availability

NanoDam data have been deposited at GEO and are publicly available as of the date of publication. Any additional information required to reanalyze the data reported in this paper is available from the lead contact upon request. Accession numbers are listed in the key resources table. All original code has been deposited at GitHub and publicly available of the date of publication. DOIs are listed in the key resources table.
